# 2-(Substituted phenyl)-3,4-dihydroisoquinolin-2-iums as Novel Antifungal Lead Compounds: Biological Evaluation and Structure-Activity Relationships

**DOI:** 10.3390/molecules180910413

**Published:** 2013-08-29

**Authors:** Zhe Hou, Rui Yang, Cen Zhang, Li-Fei Zhu, Fang Miao, Xin-Juan Yang, Le Zhou

**Affiliations:** 1 College of Science, Northwest A&F University, Yangling 712100, Shaanxi, China; E-Mails: houzhe561@sina.com (Z.H.); yrer01018@126.com (R.Y.); z84934067@126.com (C.Z.); zhao_fx0309@163.com (L.-F.Z.); 2 College of Life Science, Northwest A&F University, Yangling 712100, Shaanxi, China; E-Mail: miaofangmf@163.com

**Keywords:** 2-aryl-3,4-dihydroisoquinolin-2-ium, antifungal activity, phytopathogenic fungi, structure-activity relationship, sanguinarine, chelerythrine

## Abstract

The title compounds are a class of structurally simple analogues of quaternary benzo[*c*]phenanthridine alkaloids (QBAs). In order to develop novel QBA-like antifungal drugs, in this study, 24 of the title compounds with various substituents on the *N*-phenyl ring were evaluated for bioactivity against seven phytopathogenic fungi using the mycelial growth rate method and their SAR discussed. Almost all the compounds showed definite activities *in vitro* against each of the test fungi at 50 μg/mL and a broad antifungal spectrum. In most cases, the mono-halogenated compounds **2**–**12** exhibited excellent activities superior to the QBAs sanguinarine and chelerythrine. Compound **8** possessed the strongest activities on each of the fungi with EC_50_ values of 8.88–19.88 µg/mL and a significant concentration-dependent relationship. The SAR is as follows: the *N*-phenyl group is a high sensitive structural moiety for the activity and the characteristics and position of substituents intensively influence the activity. Generally, electron-withdrawing substituents remarkably enhance the activity while electron-donating substituents cause a decrease of the activity. In most cases, *ortha*- and *para*-halogenated isomers were more active than the corresponding *m*-halogenated isomers. Thus, the title compounds emerged as promising lead compounds for the development of novel biomimetic antifungal agrochemicals. Compounds **8** and **2** should have great potential as new broad spectrum antifungal agents for plant protection.

## 1. Introduction

Fungal plant diseases are one of the important concerns to agricultural production and food safety worldwide [1]. Phytopathogenic fungi are able to infect any tissue at any stage of plant growth, and result in severe yield losses and the quality decrease of agricultural products. In addition, many of the phytofungal pathogens may produce mycotoxins harmful to animal and human health [2]. Therefore, various plant fungicides are extensively used in current agriculture. However, many of the currently used antifungal agents have several shortcomings, such as affecting human health, environmental pollution and development of pathogen resistance [3]. Thus, the worsening problems of phytofungal disease control urgently necessitate the discovery and development of new antifungal agents for plant protection.

In the past decades, natural product-based antimicrobial agents had attracted a lot of attention from researchers owing to the fact they are perceived to have less environmental toxicity and lower mammalian toxicity [4]. Although most natural antifungal compounds are not suitable for direct or extensive application as agricultural fungicides because of their lower activity than that of artificial antimicrobial agents, limited sources or higher cost, they have been considered as ideal leads or model compounds for the development of new environmentally acceptable antimicrobial agents.

**Figure 1:**
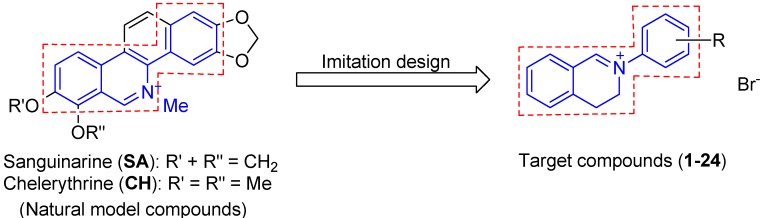
Structures of the target compounds and their model compounds.

Quaternary benzo[*c*]phenanthridine alkaloids (QBAs) are a relatively small but important class of natural isoquinolines [5,6]. Among them, sanguinarine (**SA**) and chelerythrine (**CH**) (Figure 1) are the most common. QBAs had been proven to have low toxicity to mammals [7,8,9] and extensive pharmacological activities such as antitumor [10], antimicrobial [11,12], anti-inflammatory [7], anti-HIV [13], anti-angiogenesis [14], anti-acetylcholinesterase [15] and antiparasitic actions against *Trichodina* sp. [16], *Dactylogyrus intermedius* [17], malaria [18] and *Psoroptes cuniculi* [6]. QBAs extract from plants have been used in toothpastes and mouthwashes as antiplaque agents, a veteri­nary preparation for mastoiditis in cows and an additive to animal feeds [8]. Our previous studies also demonstrated that sanguinarine and chelerythrine possessed excellent bioactivities against phytopathogenic fungi [19]. Particularly, it was worth mentioning that the iminium moiety (C=N^+^) in **SA** and **CH** had been proven to be the key structural moiety for their antimicrobial activity [12,19] and acaricidal activity [6]. The similar case was also found for their anti-tumor activity [20].

In order to develop more potent isoquinoline drugs, we designed a class of structurally simple QBAs-like compounds, *i.e.*, 2-aryl-3,4-dihydroisoquinolin-2-iums (**1**–**24**, Figure 1), based on a structural biomimetic strategy. Like QBAs, the compounds **1**–**24** possess an isoquinoline framework containing an iminium moiety (C=N^+^). Besides, the compounds have approximate equal molecular length to QBAs. We expected that the structural similarity between the designed compounds and their model compounds can lead to discovery of new compounds with higher bioactivity than the model compounds. It should be noted that unlike the complete planarity of QBAs, the compounds **1**–**24** have different stereo structures from QBAs due to the existence of a dihedral angle between two phenyl rings. The changeable dihedral angle endows the molecules with flexibility and was expected to make the molecules better match the environment of the binding site of their biotarget and display higher bioactivity.

The title compounds may be easily prepared from commercially available isochroman according to our previously reported method [21]. The reaction of isochroman with 57% hydroiodic acid under reflux yielded 1-(2-iodoethyl)-2-(iodomethyl)benzene in 96% yield. A solution of the resultant diiodide and aniline or substituted aniline in water was refluxed in the presence of sodium dodecyl sulfonate (SDS) as phase-transfer catalyst to afford the corresponding 2-aryl-1,2,3,4-tetrahydroisoquinoline in 86%–94% yield. Finally, the 2-aryl-1,2,3,4-tetrahydroisoquinolines were selectively oxidized with DDQ (2,3-dichloro-5,6-dicyano-p-benzoquinone) or CuCl_2_·2H_2_O followed by treatment of hydrobromic acid to provide the title compounds as solids in 40%–99% yield.

As a part of our ongoing program for the development of QBAs-like isoquinoline drugs, we report herein the evaluation of the antifungal activity of compounds **1**–**24** against various plant pathogenic fungi and the establishment of their structure-activity relationships. To our knowledge, there has been no information on the antifungal activity of the title compounds in the literature until now.

## 2. Results and Discussion

### 2.1. Screening of Antifungal Activity *in Vitro*

Compounds **1**–**24** were obtained by chemical synthesis according to our previous method [21] and their structures and substituent patterns are shown in Figure 1 and Table 1. According to the mycelial linear growth rate method [19], **1**–**24** were screened for antifungal activities *in vitro* against seven phytopathogenic fungi (*Alternaria alternate*, * Curvularia lunata*, *Pyricularia oryza*, *Fusarium solani*, *Valsa mali*, *Fusarium oxysporum sp. niveum* and *Fusarium oxysporum f.* sp*. vasinfectum.*) at 50 μg/mL. **SA** iodide and **CH** iodide as model compounds, and triabendazole (**TBZ**, ≥99%), a commercial fungicide standard, were used as positive controls. Mean inhibition rates of all the test compounds against the same fungus were pairwise compared by Duncan’s multiple test. The results are listed in Table 1.

**Table 1:** Substituent patterns and antifungal activities of compounds at 50 μg/mL (72 h). *

Compound		Linear growth inhibitory rates (means, %) **						
No.	R	A.A.	C.L.	P.O.	F.S.	V.M.	F.O.N.	F.O.V.
**1**	H	56.8 h	76.7 ef	50.6 hi	23.5 k	32.4 l	50.8 h	25.6 m
**2**	*o*-F	78.4 b	95.1 a	69.9 b	85.4 b	80.8 bc	80.1 c	71.3 c
**3**	*m*-F	66.4 def	86.9 bcd	62.2 cdef	48.5 ghij	54.2 i	44.1 i	43.1 h
**4**	*p*-F	78.4 b	73.4 fg	62.2 cdef	59.7 def	66.4 g	71.1 de	59.5 e
**5**	*o*-Cl	84.1 a	84.1 cd	67.9 bc	59.2 def	72.9 ef	74.0 f	55.8 f
**6**	*m*-Cl	68.3 d	84.5 cd	60.9 def	62.1 de	72.1 ef	36.6 j	34.9 ij
**7**	*p*-Cl	78.4 b	89.0 bc	64.1 bcde	55.8 defgh	61.2 g	74.0 f	65.1 d
**8**	*o*-Br	78.1 b	92.0 ab	82.3 a	93.9 a	71.7 ef	91.7 b	74.6 b
**9**	*p*-Br	72.1 c	69.4 g	62.8 cde	49.0 ghij	55.5 i	65.9 f	61.5 e
**10**	*o*-I	65.9 def	81.6 de	60.3 def	55.3 defghi	70.8 f	67.3 ef	52.8 f
**11**	*m*-I	64.2 f	84.9 cd	59.0 defg	54.3 efghi	53.3 i	28.5 k	25.6 m
**12**	*p*-I	60.6 g	73.4 fg	53.8 gh	47.0 ij	77.3 cd	44.1 i	43.6 h
**13**	*o*-CF_3_	64.0 f	73.9 fg	65.4 bcd	53.8 efghi	55.9 i	48.4 hi	48.2g
**14**	*m*-CF_3_	64.9 ef	86.1 bcd	63.5 cde	47.5 hij	47.2 j	35.1 j	31.3 kl
**15**	*p*-CF_3_	67.8 de	87.7 bcd	58.3 efg	44.6 j	38.5 k	36.1 j	33.8 jk
**16**	*o*-NO_2_	11.1 m	4.0 l	12.8 n	−12.2 n	2.7 op	5.3 m	8.2 o
**17**	*m*-NO_2_	73.1 c	73.9 fg	70.4 b	56.81 defg	47.2 j	56.9 g	52.8 f
**18**	*p*-NO_2_	63.5 f	60.8 h	62.2 cdef	58.7 def	82.3 ab	36.1 j	37.9 i
**19**	*o*-Me	51.0 i	58.7 ghi	32.7 l	−0.6 m	2.3 p	13.8 l	10.8 o
**20**	*m*-Me	11.6 m	41.6 k	19.2 m	2.6 m	11.6 n	8.1 m	11.3 o
**21**	*p*-Me	60.1 g	57.1 ghi	35.3 kl	13.5 l	6.6 o	33.2 j	21.0 n
**22**	*o*-OH	47.2 k	53.8 ij	44.9 ij	53.4 fghi	11.0 n	25.2 k	20.0 n
**23**	*p*-OH	58.2 gh	87.7b cd	55.8 fgh	73.3 c	76.0 de	35.1 j	30.8 kl
**24**	*p*-OMe	55.8 h	57.5 hi	46.8 i	22.7 k	25.0 m	36.6 j	29.7 l
**SA**	—	47.7 jk	58.7 hi	40.7 jk	56.4 defg	69.1 fg	50.5 h	59.7 e
**CH**	—	50.3 ij	48.7 j	50.1 hi	63.5 d	57.1 i	63.2 f	47.6 g
**TBZ**	—	16.4 l	9.3 l	6.4 o	81.5 b	86.0 a	100.0 a	100.0 a

* A.A., Alternaria alternate; C.L., Curvularia lunata; P.O., Pyricularia oryza; F.S., Fusarium solani; V.M., Valsa mali; F.O.N., Fusarium oxysporum sp. niveum; F.O.V., Fusarium oxysporum f. sp. vasinfectum. ** The differences between data with the different lowercase letters within a column are significant for the same tested fungus (*p* < 0.05).

The results in Table 1 show that almost all the target compounds **1**–**24** displayed activities in varying degrees against each of the test fungi at 50 μg/mL, indicating that the compounds had a wide antifungal spectrum similar to that of **SA** and **CH**. For every one of the fungi, all or some of the compounds were found to be more active than **SA**, **CH** and/or **TBZ** (*p* < 0.05). For the fungi *A. alternate*, *C. lunata* and * P. oryza*, all or most of the compounds were more active than **SA**, **CH** and/or **TBZ** (*p* < 0.05). Among them, **2**, **4**, **5**, **7** and **8** showed the greatest or greater inhibition rates (78.4%, 78.4%, 84.1%, 78.4% and 78.1%) on *A. alternate*, **2**, **3**, **7**, **8**, **14**, **15** and **23** displayed the strongest or stronger activities with inhibition rates of 95.1%, 86.9%, 89.0%, 92.0%, 86.1%, 87.7% and 87.7% on * C. lunata*, and **8** exhibited the best activity (82.3%) on *P. oryza*, followed by **2** (69.9%), **5** (67.9%), **7** (64.1%), **13** (65.4%) and **17** (70.4%).

For each of the fungi *F.*, * V. mali*, *solani*, *F. oxysporum* sp. *niveum* and * F. oxysporum* f. sp. *vasinfectum*, some of the compounds showed higher or equal activity to that of **SA**, CH or TBZ. For *F.* *solani*, 15 of the compounds showed the same or higher activities (48.5–93.9%) than that (56.4%) of **SA** while 10 compounds gave the same or higher activities (55.3–93.9%) than that (63.5%) of **CH** (*p* > 0.05). Among them, **8** and **2** gave the strongest activity (93.9%, 85.4%) over or equal to that (81.5%) of **TBZ** (*p* > 0.05). For *V. mali*, 10 of the compounds were equally or more active (53.3~82.3%) than **CH** (57.1%) and 15 of the compounds were equally or more active (61.2–82.3%) than **SA** (69.1%) (*p* > 0.05). **18** (R = *p*-NO_2_) revealed the highest activity (82.3%), equal to that of **TBZ** (86.0%) (*p* > 0.05).

As to *F. oxysporum* sp. *niveum*, 10 of the 24 compounds were equally or more active (48.4–91.7%) than **SA** (50.5%) while seven of the compounds were equally or more active than **CH** (63.2%) (*p* > 0.05). For *F. oxysporum* f. sp. *vasinfectum*, five of the compounds were equally or more active than **SA** (59.7%) while nine of the compounds were equally or more active (48.2–74.6%) than **CH** (47.6%). In both cases mentioned above, **8** revealed the highest activities (91.7%, 74.6%), but the activities of all the compounds were weaker than that of **TBZ** (100.0%).

### 2.2. Structure-Activity Relationship

**Figure 2:**
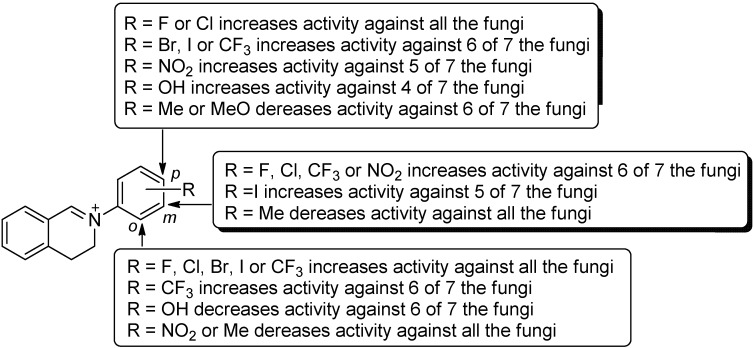
Structure-activity relationship of the compounds **1**–**24**.

Qualitative structure-activity relationship was established by comparison of both the activities and structures of various test compounds **1**–**24**. By comparison of the activity of **1** (R = H) with that of the other compounds (**2**–**24**) on each the fungus (Table 1), it was clearly seen that the introduction of various substituents to *N*-aromatic ring had significant effects on the activity, indicating that the *N*-phenyl group was a high sensitive structural moiety for the activity. The general trend was that electron-withdrawing substituents like halogen atoms (**2**–**12**), trifluoromethyl groups (-CF_3_) (**13**–**15**) and nitro groups (*m*- and *p*-NO_2_) (**17**, **18**), especially halogen atoms, remarkably enhanced the activity. On the contrary, the presence of electron-donating groups like -CH_3_ (**19**–**21**), *o*-OH (**22**), and *p*-OCH_3_ (**24**) led to a decrease of the activity in most cases (Figure 2).

On the other hand, by comparison of the activity of various isomers on each the fungus, a significant effect of the position of the substituents on the activity was observed. For halogenated isomers (**2**–**12**), the order of position effect of the substituents (-F, -Br, -Cl, -I) was *o*-substituted isomer > *p*-substituted isomer > *m*-substituted isomer in most cases. For example, for the three fluorinated compounds **2**–**4**, the *o*-F isomer **2** showed the strongest activity against each the test fungi, the *p*-F isomer **4** was the second, except for *C. lunata*, and the *m*-F isomer **3** gave the weakest activity except for *C. lunata*. A similar trend was also observed for chlorinated, brominated and iodinated isomers **5**–**12**. The case for trifluoromethyl-substituted isomers was similar, but not identical to that of halogen atoms. Like the halogenated isomers **2**–**12**, the *o*-CF_3_ isomer **13** also gave the stronger activity on most of the fungi (6/7) than the *m*-CF_3_ isomer **14** and *p*-CF_3_ isomer **15**, but the activities of **14** and **15** showed no significant difference for all tested fungi except for *V. mali* (*p* > 0.05). The results above showed that for halogen atoms and trifluoromethyl group, the 2-site of the *N*-phenyl ring was beneficial for the improvement of the activity. Unlike the cases of halogen atoms and -CF_3_ group, an *o*-nitro group (compound **16**) caused an obvious decrease of the activity for all the fungi while a *m*-nitro group (compound **17**) and *p*-nitro group (compound **18**) led to an enhancement of the activity. Among them, the *m*-nitro-substituted isomer **17** showed the best activity on 6 of 7 the fungi.

The results above indicate that the activity of the target compounds greatly depended on the characteristic and position of substituents on the *N*-aromatic ring. Generally, the introduction of F, Cl or Br to the I site in the *N*-phenyl ring resulted in a very great improvement of the activity.

### 2.3. Antifungal Toxicity

In order to more fully understand the antifungal activities of the compounds, the most active compounds **2**, **4**, **5**, **7** and **8** were further evaluated as representative compounds for antifungal toxicity on seven phytophathogenic fungi.

**Table 2:** Toxicity regression equations and EC_50_ values of **2**, **4**, **5**, **7** and **8** against 7 fungi.

Fungus	Compd.	Toxicity regression equation *	*R^2^*	EC_50_		95%CI ** of EC_50_
				(μg/mL)	(μmol/L)	
F.O.V.	**2** (*o*-F)	*y =* 1.4300*x* + 3.0769	0.9780	22.12	72.2	20.74–23.60
	**4** (*p*-F)	*y =* 2.6168*x* + 0.4853	0.9381	53.12	173.5	42.10–67.02
	**5** (*o*-Cl)	*y =* 1.2450*x* + 3.0099	0.9433	39.67	123.0	36.31–72.17
	**7** (*p*-Cl)	*y =* 2.3044*x* + 1.3235	0.9971	39.39	122.1	39.29–40.05
	**8** (*o*-Br)	*y =* 1.0996*x* + 3.7981	0.9958	12.39	33.8	12.09–12.70
	**SA**	*y =* 1.6623*x* + 2.4605	0.9730	33.71	73.4	23.26–48.85
	**CH**	*y =* 3.0902*x* − 0.3177	0.9737	52.58	110.6	29.43–93.95
F.O.N.	**2** (*o*-F)	*y =* 2.4275*x* + 1.3864	0.9769	30.80	100.6	29.98–31.64
	**4** (*p*-F)	*y =* 2.4139*x* + 1.5676	0.9669	26.42	86.3	25.36–27.52
	**5** (*o*-Cl)	*y =* 1.3796*x* + 3.2094	0.9373	19.86	61.6	18.05–37.91
	**7** (*p*-Cl)	*y =* 2.7227*x* + 0.9408	0.9988	30.97	96.0	30.88–31.06
	**8** (*o*-Br)	*y =* 1.8107*x* + 3.1516	0.9414	10.49	28.6	7.48–14.71
	**SA**	*y =* 1.4175*x* + 2.5893	0.9853	50.20	109.3	43.44–58.01
	**CH**	*y =* 1.7052*x* + 2.5090	0.9864	28.89	60.8	23.77–35.12
V.M.	**2** (*o*-F)	*y =* 2.3031*x* + 1.8962	0.9836	22.27	72.7	21.71–26.42
	**4** (*p*-F)	*y =* 2.3521*x* + 1.3300	0.9162	36.33	118.7	33.79–59.37
	**5** (*o*-Cl)	*y =* 2.0946*x* + 2.0847	0.9651	24.65	76.4	23.21–26.18
	**7** (*p*-Cl)	*y =* 1.4730*x* + 2.9048	0.9749	26.45	82.0	25.87–30.72
	**8** (*o*-Br)	*y =* 1.1731*x* + 3.4768	0.9575	19.88	54.2	16.03–24.66
	**SA**	*y =* 1.9432*x* + 2.2639	0.9880	25.59	55.7	17.19–38.09
	**CH**	*y =* 1.9658*x* + 1.8575	0.9541	39.68	83.5	16.17–97.36
F.S.	**2** (*o*-F)	*y =* 2.1583*x* + 2.0918	0.9938	22.25	72.7	22.05–23.64
	**4** (*p*-F)	*y =* 1.2769*x* + 3.4716	0.9846	15.74	51.4	15.52–17.30
	**5** (*o*-Cl)	*y =* 1.0950*x* + 3.4884	0.9583	24.02	74.5	22.95–32.70
	**7** (*p*-Cl)	*y =* 0.9805*x* + 3.5004	0.9534	33.84	104.9	29.63–38.65
	**8** (*o*-Br)	*y =* 2.1420*x* + 2.9683	0.9833	8.88	24.2	8.04–9.81
	**SA**	*y =* 2.1928*x* + 1.4479	0.9893	41.68	90.8	31.65–54.89
	**CH**	*y =* 1.3614*x* + 3.0620	0.9466	26.52	55.8	15.92–44.19
A.A.	**2** (*o*-F)	*y =* 1.3559*x* + 3.2997	0.9835	17.95	58.6	17.50–21.32
	**4** (*p*-F)	*y =* 2.6327*x* + 1.3957	0.9876	23.39	76.4	22.96–26.56
	**5** (*o*-Cl)	*y =* 1.1348*x* + 3.5676	0.9738	18.29	56.7	17.57–24.00
	**7** (*p*-Cl)	*y =* 1.3719*x* + 3.3852	0.9813	15.03	46.6	14.60–18.29
	**8** (*o*-Br)	*y =* 1.1204*x* + 3.9040	0.9953	9.51	25.9	9.25–9.78
	**SA**	*y =* 2.2140*x* + 1.1814	0.9550	53.03	115.5	28.22–99.67
	**CH**	*y =* 2.6508*x* + 0.5078	0.9586	49.51	104.2	21.52–113.92
C.L.	**2** (*o*-F)	*y =* 1*.*3293x + 3.6629	0.9725	10.14	33.1	7.28–14.11
	**4** (*p*-F)	*y =* 1*.*0827*x* + 3*.*7694	0.9198	13.70	44.7	12.10–31.73
	**5** (*o*-Cl)	*y =* 1.3088*x* + 3*.*7443	0.9169	9.11	28.2	7.10–11.69
	**7** (*p*-Cl)	*y =* 1.4152*x* + 3.4244	0.9675	12.98	40.2	12.33–18.34
	**8** (*o*-Br)	*y =* 1.7125*x* + 3.3707	0.9373	8.94	24.4	6.38–12.53
	**SA**	*y* = 1.5699*x* + 2.5652	0.9621	35.55	77.4	23.37–54.07
	**CH**	*y =* 0.9154*x* + 3.9048	0.9621	15.72	33.1	6.08–40.66
P.O.	**2** (*o*-F)	*y =* 1.0351*x* + 3.7446	0.9531	16.33	53.3	15.63–21.97
	**4** (*p*-F)	*y =* 1.4434*x* + 2.8066	0.9892	33.08	108.0	32.78–35.15
	**5** (*o*-Cl)	*y =* 0.8119*x* + 3.9161	0.9818	21.63	67.0	20.66–22.82
	**7** (*p*-Cl)	*y =* 1.6338*x* + 2.6110	0.9893	28.99	89.9	28.73–30.81
	**8 **(*o*-Br)	*y =* 1.4194*x* + 3.4358	0.9806	12.65	34.46	11.15–14.35
	**SA**	*y* = 0.8326*x* + 3.3309	0.9754	101.09	220.1	72.61–140.73
	**CH**	*y =* 1.6792*x* + 2.1968	0.9629	46.71	98.3	39.01–55.93

* *y*: The probability of the inhibition rate; *x*: log[concentration (*μ*g/mL)]. ** 95% CI: Lower and upper values of the confidence interval of EC_50_ (*μ*g/mL) at 95% probability.

Meanwhile, both **SA** and **CH** were used as positive controls. Based on the mycelial growth inhibitory rates of the compounds at a series of concentrations, toxicity regression equations of the compounds were established between log[concentration] value and probit value of inhibition rates by using the least square method. EC_50_ (median effective concentration) values of the compounds and their confidence intervals at 95% probability were calculated from the corresponding toxicity regression equations. The results are listed in Table 2.

All the tested compounds showed significant concentration-dependent antifungal activity toward every one of the tested fungi (*R*^2^ values = 0.9162–0.9988) (Table 2) and their EC_50_ values were in a range of 8.88 to 53.12 µg/mL. From Table 2, it was clearly seen that most of the EC_50_ values (27/35) of the tested compounds were lower than 30 µg/mL. By contrast, most of EC_50_ values (10/14) of **SA** and **CH** were greater than 30 µg/mL. Among the tested compounds, **8** possessed the strongest activity on each the fungi and most of its EC_50_ values (6/7) were lower than 15 µg/mL. Therefore, as far as the EC_50_ values were concerned, **2**, **4**, **5**, **7** and **8** possessed stronger activity than **SA** and **CH** in most cases. In addition, It was worth mentioning that all the test compounds showed the highest activity on *C. lunata* (EC_50_ = 8.94–13.7 µg/mL) among the seven fungi.

A slope value (*k*) in a toxicity regression equation reflects concentration effect of a compound on its bioactivity. A greater k value implies that the antifungal activity of a test compound is more susceptible to its concentration change. The results in Table 2 showed that the *k* values of each the test compound on various fungi or the *k* values of various compounds on the same fungus were different, indicating that both the concentration effect of each the compound on the various fungi and the concentration effect of the various compounds on the same fungus were different.

**Figure 3:**
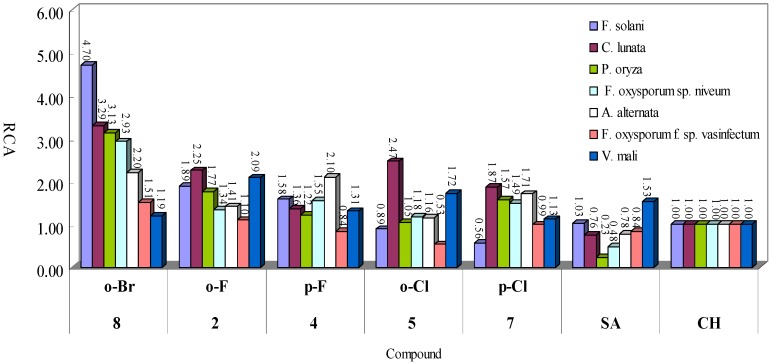
Relative comprehensive activities of the compounds against seven fungi.

A compound with better bioactivity should have a greater slope value and a smaller EC_50_ value at the same time. The ratio value of *k*/EC_50_ of a compound reflects its comprehensive activity (CA) to certain degree. In order to conveniently compare the CAs of the various compounds, we calculated the ratio of CA of each the compound to that of **CH** for the same fungus, *i.e.*, relative comprehensive activity (RCA). As shown in Figure 3, 30 of 35 the RCA values of the five test compounds were greater than 1.0, *i.e.*, the RCA value of **CH**. This result indicated that all the tested compounds possessed stronger RCA than **CH** in most cases, in agreement with the data obtained from EC_50_ values in Table 2. In particular, both **2** and **8** (RCA = 1.10–4.70) showed higher activity than **CH** against all the tested fungi. Among the tested compounds, **2** showed the strongest activity against *V. mail* while **8** showed the strongest activity against the other fungi.

## 3. Experimental

### 3.1. General

Compounds **1**–**24** were obtained by synthesis according to our previously reported methods and their structures were determined by spectroscopic analysis [21]. Sanguinarine iodide (**SA**, >98%) and chelerythrine iodide (**CH**, >99%) were obtained in our laboratory by isolation from the whole plant of *M.* *microcarpa* (Maxim) Fedde [12]. Thiabendazole (**TBZ**, ≥99.1%), a commercial fungicide standard, was purchased from Sigma-Aldrich Trading Co. Ltd. (Shanghai, China). Dimethyl sulfoxide (DMSO) was purchased from J&K Chemical Ltd. (Beijing, China). Other reagents were obtained locally and of analytical grade. The water used was redistilled and ion-free.

Test fungi *Fusarium oxysporum* sp. vasinfectum, *Fusarium oxysporum* sp. niveum, *Valsa mali*, *Fusarium solani*, *Alternaria alternate*, *Curvularia lunata* and *Pyricularia oryzae* were isolated, identified and provided by the Center of Pesticide Research, Northwest A&F University, Yangling, China. These fungi were grown on potato-dextrose-agar (PDA) plates at 28 °C and maintained at 4 °C by periodic subculturing.

### 3.2. Assay of Antifungal Activity *in vitro*

The antifungal activity *in vitro* was assayed by the growth rate method reported by us [19]. The test fungi maintained on PDA medium slants were subcultured for 48 h in Petri dishes prior to testing and used for inoculation of fungal strains on PDA plates. The tested compounds were completely dissolved in DMSO, and then diluted by water to provide a stock solution (1.2 mg/mL) in 5% DMSO aqueous solution. Thiabendazole solution (1.2 μg/mL) in 5% DMSO aqueous solution and 5% DMSO aqueous solution were used as positive drug control and blank control, respectively. **SA** and **CH** solutions (1.2 μg/mL) in 5% DMSO aqueous solution were used as the model compound controls at the same time. The stock solution (10 mL) was completely mixed with the autoclaved PDA medium (230 mL) to provide a medium containing 50 μg/mL of test compounds and then poured into the Petri dishes in a laminar flow chamber. When the medium in the plate was partially solidified, a 5-mm thick and 4-mm diameter disc of fungus cut from earlier subcultured Petri dishes was placed at the centre of the semi-solid medium. The dishes were kept in an incubator at 28 °C for 72 h. Each experiment was carried out in triplicate. The diameters (in mm) of inhibition zones were measured in three different directions and the growth inhibition rates were calculated according to the following formula and expressed as means ± S.D.:

Growth inhibition rate (%) = [(*d*_c_ − *d*_0_) − (*d*_s_ − *d*_0_)]/(*d*_c_ − *d*_0_) × 100 (1)

where *d*_0_: Diameter of the fungus cut, *d*_c_: Diameter of the blank control fungus, *d*_s_: Diameter of the compound-treated fungus.

Based on the *in vitro* antifungal activity screening results, the more active compounds **2**, **4**, **5**, **7** and **8** were selected to determine their antifungal toxicity according to the method described above [19]. A stock solution of the compounds was prepared in 5% DMSO aqueous solution, and then diluted by 5% DMSO in water using serial two-fold dilution method to obtain a series of stock solutions. Each stock solution (10 mL) was respectively mixed with the autoclaved PDA medium to prepare a set of media containing 120, 90, 80, 40, 20, 10, 5 and 2.5 μg/mL compounds. Meanwhile, 5% DMSO aqueous solution was used as blank control. The antifungal activity for each concentration of the compounds was determined. Each experiment was performed in triplicate. The average inhibition rate for each test was calculated and then transformed to the corresponding probit value. The concentration of the compound was transformed to the corresponding logarithm value (log_10_*C*). Log_10_*C* values for each compound and its corresponding probit values were used to establish toxicity regression equation by using the least square method. EC_50_ values and their confidence interval at 95% probability were calculated from the toxicity regression equations. RCA of each compound was calculated according to the following formula:

RCA = (slope value of the test compound/slope value of **CH**) × (EC_50_ value of **CH**/EC_50_ value of the test compound) (2)

### 3.3. Statistic Analysis

SPSS V17.0 statistical software was used to analyze the data and establish toxicity regression equations. Duncan multiple comparison test was performed on the data to determine significant difference between the inhibition rates of various compounds at the same concentration.

## 4. Conclusions

2-Aryl-3,4-dihydroisoquinolin-2-ium bromides are a class of structurally simple QBA-like compounds. In the present study, we evaluated *in vitro* antifungal activities of a series of synthesized 2-aryl-3,4-dihydroisoquinolin-2-ium bromides with various substituents on the *N*-aromatic ring against seven pathogenic fungi and discussed their structure-activity relationships as well as compared their activities with that of their natural model compounds **SA** and **CH**. Like **SA** and **CH**, almost all the compounds **1**–**24** displayed the activities against all the fungi in varying degrees at 50 μg/mL and a broad antifungal spectrum. The *N*-phenyl ring was proven to be a highly influential structural moiety for the activity. The introduction of substituents to *N*-phenyl ring led to significant change of the activity. Generally, electron-withdrawing substituents such as -X (X = F, Cl, Br or I), -CF_3_ and -NO_2_, especially -X, remarkably enhanced the activity. On the contrary, electron-donating groups such as -CH_3_, *o*-OH, and *p*-OCH_3_ result in the decrease of the activity in most cases. In addition, the position of substituents was able to significantly influence the activity also. For halogenated compounds, the order of the activity of various isomers was generally *o*-substituted isomer > *p*-substituted isomer > *m*-substituted isomer. In most cases, most of the halogenated compounds **2**~**12** showed higher activity than **SA** and **CH**. **2**, **4**, **5**, **7** and **8** showed significant concentration-dependent antifungal activity toward the tested fungi. Both **2** and **8**, especially **8**, showed higher RCAs than **SA** and **CH** against all the 7 tested fungi. Thus, 2-aryl-3,4-dihydroisoquinolin-2-ium compounds might be considered as promising lead compounds for the development of novel isoquinoline antifungal agrochemicals. Compounds **2**, **4**, **5**, **7** and **8**, and especially **2** and **8**, are of great potential as new antifungal agents for plant protection.

Compared with the natural model compounds **SA** or **CH**, the title compounds had some obvious advantages such as more simple structure, higher bioactivity, higher high atom economy, easy chemical synthesis on a large scale and lower cost, *etc.* In addition, our recent research had demonstrated that the title compounds were safe for plant growth (unpublished data). Therefore, the title compounds should have broad application prospects in agriculture as novel biomimetic fungicides and alternatives to the natural model compounds.

## References

[1] Savary S., Teng P.S., Willocquet L., Nutter F.W. (2006). Quantification and modeling of crop losses: A review of purposes. Annu. Rev. Phytopathol..

[2] Bräse S., Encinas A., Keck J., Nising C.F. (2009). Chemistry and biology of mycotoxins and related fungal metabolites. Chem. Rev..

[3] Bai Y.B., Zhang A.L., Tang J.J., Gao J.J. (2013). Synthesis and antifungal activity of 2-chloromethyl-1H-benzimidazole derivatives against phytopathogenic fungi *in vitro*. J. Agric. Food Chem..

[4] Wedge D.E., Camper N.D., Cutler H.G., Cutler S.J. (2000). Biologically active natural products. Agrochemicals and Pharmaceuticals.

[5] Dostál J, Slavík J., Atta-ur-Rahman (2002). Some aspects of the chemistry of quaternary benzo[*c*]phenanthridine alkaloids. Studies in Natural Products Chemistry.

[6] Miao F., Yang X.J., Ma Y.N., Zheng F., Song X.P., Zhou L. (2012). Structural modification of sanguinarine and chelerythrine and their *in vitro* acaricidal activity against *Psoroptes cuniculi*. Chem. Pharm. Bull..

[7] Lenfeld J., Kroutil M., Maršálek E., Slavík J., Preininger V., Šimánek V. (1981). Antiinflammatory activity of quaternary benzophenanthridine alkaloids from *Chelidonium majus*. Planta Med..

[8] Psotova J., Vecera R., Zdarilova A., Anzenbacherova E., Kosina P., Svobodova A., Hrbac J., Jirovsky D., Stiborova M., Lichnovsky V. (2006). Safety assessment of sanguiritrin, Alkaloid fraction of *Macleaya cordata*, in rats. Vet. Med..

[9] Kosina P., Walterova D., Ulrichova J., Lichnovsky V., Stiborova M., Rydlova H., Vicar J., Krecman V., Brabec M.J., Simanek V. (2004). Sanguinarine and chelerythrine: Assessment of safety on pigs in ninety days feeding experiment. Food Chem. Toxicol..

[10] Ahsan H., Reagan-Shaw S., Breur J., Ahmad N. (2007). Sanguinarine induces apoptosis of human pancreatic carcinoma AsPC-1 and BxPC-3 cells via modulations in Bcl-2 family proteins. Cancer Lett..

[11] Meng F.Y., Zuo G.Y., Hao X.Y., Wang G.C., Xiao H.T., Zhang J.Q., Xu G.L. (2009). Antifungal activity of the benzo[*c*]phenanthridine alkaloids from *Chelidonium majus* Linn against resistant clinical yeast isolates. J. Ethnopharmacol..

[12] Miao F., Yang X.J., Zhou L., Hu H.J., Zheng F., Ding X.D., Sun D.M., Zhou C.D., Sun W. (2011). Structural modification of sanguinarine and chelerythrine and their antibacterial activity. Nat. Prod. Res..

[13] Kerry M.A., Duval O., Waigh R.D., Mackay S.P. (1998). The role of the iminium bond in the inhibition of reverse transcriptase by quaternary benzophenanthridines. J. Pharm. Pharmacol..

[14] Eun J.P., Koh G.Y. (2004). Suppression of angiogenesis by the plant alkaloid, Sanguinarine. Biochem. Biophys. Res. Commun..

[15] Cho K.M., Yoo I.D., Kim W.G. (2006). 8-Hydroxydihydrochelerythrine and 8-hydroxydihydrosanguinarine with a potent acetylcholinesterase inhibitory activity from *Chelidonium majus* L.. Biol. Pharm. Bull..

[16] Yao J.Y., Li X.L., Shen J.-Y., Pan X.Y., Hao G.J., Xu Y., Ying W.L., Ru H.S., Liu X.L. (2011). Isolation of bioactive components from *Chelidonium majus* L. with activity against *Trichodina* sp.. Aquaculture.

[17] Wang G.X., Zhou Z., Jiang D.X., Han J., Wang J.F., Zhao L.W., Li J. (2010). *In vivo* anthelmintic activity of five alkaloids from *Macleaya microcarpa* (Maxim) Fedde against *Dactylogyrus intermedius* in *Carassius auratus*. Vet. Parasitol..

[18] Nyangulu J.M., Hargreaves S.L., Sharples S.L., Mackay S.P., Waigh R.D., Duval O., Mberu E.K., Watkins W.M. (2005). Antimalarial benzo[*c*]phenanthridines. Bioorg. Med. Chem. Lett..

[19] Yang X.J., Miao M., Yao Y., Cao F.J., Yang R., Ma Y.N., Qin B.F., Zhou L. (2012). *In vitro* antifungal activity of sanguinarine and chelerythrine derivatives against phytopathogenic fungi. Molecules.

[20] Nakanishi T., Suzuki M., Saimoto A., Kabasawa T.J. (1999). Structural considerations of NK109, An antitumor benzo[*c*]phenanthridine alkaloid. J. Nat. Prod..

[21] Ma Y.N., Yang X.J., Pan L., Hou Z., Geng H.L., Song X.P., Zhou L., Miao F. (2013). Synthesis of 2-aryl-3,4-dihydroisoquinolin-2-ium bromides and their *in vitro* acaricidal activity against *Psoroptes cuniculi*. Chem. Pharm. Bull..

